# The magneto-microbiome: A dataset of the metagenomic distribution of magnetotactic bacteria

**DOI:** 10.1016/j.dib.2024.110073

**Published:** 2024-01-18

**Authors:** Robert R. Fitak

**Affiliations:** Department of Biology, Genomics and Bioinformatics Cluster, University of Central Florida, Orlando, FL 32816, USA

**Keywords:** Next-generation sequencing, Magnetite, Magnetosome, Sequence read archive

## Abstract

Magnetotactic bacteria (MTB) are diverse prokaryotes characterized by their ability to generate biogenic magnetic iron crystals. MTB are ubiquitous across aquatic environments, and growing evidence has indicated they may be present in association with animal microbiomes. Unfortunately, they are difficult to culture *in vitro* and more studies understanding their biogeographical distribution and ecological roles are needed. To provide data regarding the patterns of diversity and distribution of MTB, we screened the entire Sequence Read Archive (SRA) from the National Center for Biotechnology Information for DNA sequencing reads matching known MTB taxa. The dataset summarizes the count of reads assigned to MTB from more than 26 million SRA accessions comprising approximately 80 petabases (7.98 × 10^16^) of DNA. More than 396 million DNA sequencing reads were assigned to 214 MTB taxa in 691,086 (2.65 %) SRA accessions. The final dataset can be utilized by researchers to narrow their efforts in examination of both environmental and ecological roles of specific MTB or to identify potential host organisms. These data will be instrumental to further elucidating the importance and utility of these enigmatic bacteria.

Specifications Table

**Table:** 

Subject	Biological Sciences
Specific subject area	Microbiology: Microbiome; Environmental Genomics and Metagenomics
Type of data	Tables
How the data were acquired	Data were acquired by processing the entire Sequence Read Archive (SRA) from the National Center for Biotechnology Information (NCBI) for reads matching known magnetotactic bacteria using the Google BigQuery cloud environment. Known magnetotactic bacteria were queried according to their NCBI taxonomy accession and counts of all next-generation sequencing reads in the entire publicly available SRA that could be unambiguously assigned to these taxa were obtained. The data include a markdown-formatted PDF file, *Methods.pdf*, containing all the computational code and associated annotations required to generate the dataset.
Data format	Analyzed Filtered Comma-separated values
Description of data collection	The number of next-generation DNA sequencing reads that could be taxonomically assigned to known species of magnetotactic bacteria was obtained from the entire SRA database. The counts of reads were summarized for each accession in the SRA database and divided among the taxonomic origin of the original DNA sequencing dataset.
Data source location	The primary data are the entire contents of the Sequence Read Archive (SRA) from the National Center for Biotechnology Information (NCBI) on June 15, 2023.
Data accessibility	Repository name: Sequence Read Archive; for all original, primary data Direct URL to data: https://www.ncbi.nlm.nih.gov/sra Repository name: Mendeley Data (secondary data) Data identification number: 10.17632/pxvd47zxtz.12 Direct URL to data: https://data.mendeley.com/datasets/pxvd47zxtz/12

## 1 Value of the Data

• These data are useful for understanding the spatiotemporal distribution and diversity of magnetotactic bacteria across environments and hosts.
• Researchers who study the biogeography and evolutionary ecology of microbial magnetotaxis and the role of the microbiome in animal sensory physiology will benefit from the availability of these data.
• These data can be used to identify and prioritize environments or host species for future studies of the ecological role of magnetotactic bacteria or their potential for contributing geomagnetic information to their host. These data can also be used for designing experiments that target the impacts of specific species of magnetotactic bacteria or their collective diversity on host physiology.

## 2 Objective

Magnetotactic bacteria (MTB) are a diverse group of prokaryotes that biomineralize iron to form nano-sized magnetic crystals that are stored in a unique organelle called the magnetosome [1,2]. By generating chains of magnetic particles, MTB can passively align with magnetic fields (i.e., magnetotaxis). The magnetosomes and magnetic properties of MTB have made them especially useful in numerous biomedical, technological, and engineering applications such as drug delivery, magnetic resonance imaging contrast agents, printing toner, heavy metal recovery, robotics, and astrobiology [2,3]. MTB are ubiquitous across aquatic environments and thrive at the oxic-anoxic interface, thus making them challenging to culture in the laboratory [1,2]. Recently, however, there is accumulating evidence that MTB are present in the microbiomes of many organisms, and even contribute to the ability of animals to perceive the geomagnetic field through symbiotic mechanisms [4], [5], [6], [7]. In order to better understand the ecological roles of these MTB in both the environment and in association with animal microbiomes (i.e., the magneto-microbiome), metagenomic datasets of MTB presence and biogeographical distribution across both environments and hosts are needed.

## 3 Data Description

The Sequence Read Archive (SRA) held at the National Center for Biotechnology Information (NCBI) was first established in 2009 to publicly host the explosive growth in open-access next-generation DNA sequencing data [8]. The goals of the SRA are to i) make publicly funded research data findable, accessible, interoperable and reusable (i.e., FAIR) and ii) promote novel opportunities for scientific studies that utilize the massive scale of these genetic datasets [8]. The SRA has recently been made accessible for large-scale, interactive queries through multiple cloud environments, such as the Google Cloud Platform BigQuery, and now includes the taxonomic assignment of every DNA read submitted via the SRA Tax Analysis Tool [8,9]. The data described below mined and summarized the entire set of SRA accessions for the presence of specific MTB taxa and not necessarily the presence of magnetotaxis abilities.

**Fig. 1:**
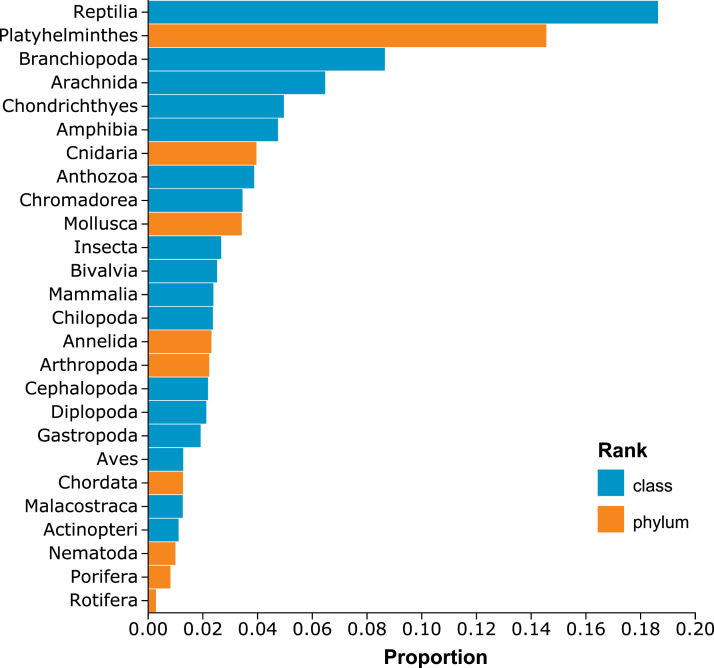
Proportion of Sequence Read Archive accessions that contain magnetotactic bacteria among various metazoan phyla and classes.

The SRA is constantly receiving new submissions, so the data described herein are from the SRA contents as of June 15, 2023 [10]. At this time, 26,126,445 SRA accessions were queried, including 359 trillion reads totaling ∼80 petabases (7.98 × 10^16^) of DNA data. A total of 691,086 (2.65 %) SRA accessions contained at least one read taxonomically assigned to a known MTB and 396,203,026 MTB-assigned reads were identified. The results of the recovered MTB reads are reported by SRA accession and by taxon in the compressed, comma-separated value (i.e., csv) spreadsheet *bq-results-20230615-225959-1686880934964.csv.gz*. A list of known MTB taxa queried according to their NCBI Taxonomy accession number (TaxIDs) is included in the spreadsheet file *MTB_taxon_sheet.xlsx*. See “**Experimental design, materials and methods**” below for a complete description of the TaxIDs. The complete dataset is available in the compressed csv spreadsheet *SRA-metatable_6-17-23_with-MTB-counts-ordered-and-taxons.csv.gz*. Because the complete dataset is quite large (> 2 GB compressed), 26 additional subsets of the dataset are available and split according to common metazoan animal phyla (nine subsets) and classes (17 subsets) to facilitate future studies of the magneto-microbiome (Fig. 1).

All the provided spreadsheets contain both the “total_count” and “self_count” for each MTB taxon, as well as the cumulative count of all MTB in the SRA accession. According to Katz, Shutov, Lapoint, Kimelman, Brister and O'Sullivan [8], the “total_count” is the sum of all reads assigned to the specific MTB taxon and all its descendent nodes in the taxonomic lineage, whereas the “self_count” is the count of reads strictly assigned the taxon listed. The SRA accessions in each spreadsheet are ordered according to decreasing cumulative MTB “self_count” content. Also available within each spreadsheet is the full taxonomic lineage, or ranks, of the origin of the specific SRA accession, including environmental and metagenomic samples, to facilitate examination by sample origin rather than MTB content.

Additional files in the dataset include a text file *README.txt* that summarizes all files in the dataset, their formats, and a description of all column headers, a markdown-generated pdf document *Methods.pdf* that details all the computer code necessary to recreate the dataset, and a list of the MD5 checksums for each file, *MD5.txt,* for users to confirm that the files have been downloaded and copied correctly.

## 4 Experimental Design, Materials and Methods

A list of known MTB taxa was obtained from existing literature [1,2,11]. Because MTB are diverse, polyphyletic, and often not culturable, this list of putative MTB is not exhaustive. Additionally, because magnetosomes can be gained and lost, the data only indicate the presence of the species listed and not necessarily the presence of species performing magnetotaxis. These taxa were subsequently identified in the NCBI Taxonomy database [12] to obtain accession numbers, hereafter *TaxIds*. The taxonomic lineages, or ranks, of these MTB TaxIds were curated manually to obtain genera, families, and orders that are specific to MTB. In other words, these higher-level taxonomic ranks solely contain lower-level TaxIds that are unique to known MTB. A total of 214 TaxIds were collected and are summarized in Table 1. Seventeen of the 214 TaxIds contained no rank and represented primarily unclassified MTB from environmental samples.

**Table 1:** Count of the MTB-specific taxa collected from the NCBI Taxonomy database. The asterisk indicates that these 17 taxa were described as having “no rank” but yet were unambiguously MTB.

Rank	Count of MTB TaxIds
Order	1
Family	2
Genus	14
Species	168
Strain	11
None*	17
**Total**	**214**

Next, the counts of next-generation sequencing reads were obtained from each accession in the NCBI SRA database that were assigned to these TaxIds [8]. To perform this search, the Google Cloud Platform BigQuery (https://cloud.google.com/bigquery) was utilized. In BigQuery, the SRA cloud dataset “*nih-sra-datastore***”** was first linked to the project. Next, a single search was performed using standard SQL syntax (see the *Methods.pdf* file in the published dataset for the full SQL script) on June 15, 2023 for the 214 TaxIds. The query processed 364.5 GB of cloud memory and took a total of 28 s. A second query was then initiated to gather the metadata for every SRA accession in the *nih-sra-datastore*. This query processed 5.96 GB of cloud memory and took 40 s to complete. This dataset was stored as 140 csv files in a “bucket” in Google's cloud services, then downloaded, merged, and compressed into a single file using the “gsutil” function in Google's Cloud SDK tools (https://cloud.google.com/sdk). The results from the two queries were merged using the *data.table v1.14.9* package [13] in R v4.2.1 [14]. Finally, taxonomic ranks were obtained and added for the origin of each SRA accession using the *taxize v0.9.100* package [15] in R, and subsets of the dataset were generated for nine common metazoan phyla and 17 classes.

## CRediT authorship contribution statement

**Robert R. Fitak:** Conceptualization, Methodology, Software, Validation, Data curation, Investigation, Writing – original draft, Writing – review & editing.

## Data Availability

The magneto-microbiome: a dataset of the metagenomic distribution of magnetotactic bacteria (Original data) (Mendeley Data)

## References

[1] Lefevre C.T., Bazylinski D.A. (2013). Ecology, diversity, and evolution of magnetotactic bacteria. Microbiol. Mol. Biol. Rev..

[2] Yan L., Zhang S., Chen P., Liu H.T., Yin H.H., Li H.Y. (2012). Magnetotactic bacteria, magnetosomes and their application. Microbiol. Res..

[3] Mathuriya A.S. (2016). Magnetotactic bacteria: nanodrivers of the future. Crit. Rev. Biotechnol..

[4] Natan E., Fitak R.R., Werber Y., Vortman Y. (2020). Symbiotic magnetic sensing: raising evidence and beyond. Philos. Trans. R. Soc. Lond. B. Biol. Sci..

[5] Natan E., Vortman Y. (2017). The symbiotic magnetic-sensing hypothesis: do magnetotactic bacteria underlie the magnetic sensing capability of animals?. Movem. Ecol..

[6] Monteil C.L., Vallenet D., Menguy N., Benzerara K., Barbe V., Fouteau S., Cruaud C., Floriani M., Viollier E., Adryanczyk G., Leonhardt N., Faivre D., Pignol D., López-García P., Weld R.J., Lefevre C.T. (2019). Ectosymbiotic bacteria at the origin of magnetoreception in a marine protist. Nat. Microbiol..

[7] Dufour S.C., Laurich J.R., Batstone R.T., McCuaig B., Elliott A., Poduska K.M. (2014). Magnetosome-containing bacteria living as symbionts of bivalves. ISME J..

[8] Katz K., Shutov O., Lapoint R., Kimelman M., Brister J.R., O'Sullivan C. (2022). The sequence read archive: a decade more of explosive growth. Nucl. Acid. Res..

[9] Katz K.S., Shutov O., Lapoint R., Kimelman M., Brister J.R., O'Sullivan C. (2021). STAT: a fast, scalable, MinHash-based k-mer tool to assess sequence read archive next-generation sequence submissions. Genom. Biol..

[10] Fitak R. (2023).

[11] Lin W., Zhang W., Zhao X., Roberts A.P., Paterson G.A., Bazylinski D.A., Pan Y. (2018). Genomic expansion of magnetotactic bacteria reveals an early common origin of magnetotaxis with lineage-specific evolution. ISME J..

[12] C.L. Schoch, S. Ciufo, M. Domrachev, C.L. Hotton, S. Kannan, R. Khovanskaya, D. Leipe, R. McVeigh, K. O'Neill, B. Robbertse, S. Sharma, V. Soussov, J.P. Sullivan, L. Sun, S. Turner, I. Karsch-Mizrachi, NCBI Taxonomy: a comprehensive update on curation, resources and tools, Database (Oxford) 2020 (2020) baaa062. 10.1093/database/baaa062. PMC7408187 32761142

[13] T. Barrett, M. Dowle, A. Srinivasan, J. Gorecki, M. Chirico, T. Hocking, Rdatatable/data.table: Extension of `data.frame'. version 1.14.9, 2023 https://Rdatatable.gitlab.io/data.table, https://github.com/Rdatatabe/data.table, https://r-datatable.com R package.

[14] R Core Development Team (2022). https://www.R-project.org/.

[15] Chamberlain S., Szöcs E. (2013). taxize: taxonomic search and retrieval in R. F1000Resarch.

